# Reading Direction and the Central Perceptual Span in Urdu and English

**DOI:** 10.1371/journal.pone.0088358

**Published:** 2014-02-25

**Authors:** Kevin B. Paterson, Victoria A. McGowan, Sarah J. White, Sameen Malik, Lily Abedipour, Timothy R. Jordan

**Affiliations:** College of Medicine, Biological Sciences and Psychology, University of Leicester, Leicester, United Kingdom; New York University, United States of America

## Abstract

**Background:**

Normal reading relies on the reader making a series of saccadic eye movements along lines of text, separated by brief fixational pauses during which visual information is acquired from a region of text. In English and other alphabetic languages read from left to right, the region from which useful information is acquired during each fixational pause is generally reported to extend further to the right of each fixation than to the left. However, the asymmetry of the perceptual span for alphabetic languages read in the opposite direction (i.e., from right to left) has received much less attention. Accordingly, in order to more fully investigate the asymmetry in the perceptual span for these languages, the present research assessed the influence of reading direction on the perceptual span for bilingual readers of Urdu and English.

**Methods and Findings:**

Text in Urdu and English was presented either entirely as normal or in a gaze-contingent moving-window paradigm in which a region of text was displayed as normal at the reader's point of fixation and text outside this region was obscured. The windows of normal text extended symmetrically 0.5° of visual angle to the left and right of fixation, or asymmetrically by increasing the size of each window to 1.5° or 2.5° to either the left or right of fixation. When participants read English, performance for the window conditions was superior when windows extended to the right. However, when reading Urdu, performance was superior when windows extended to the left, and was essentially the reverse of that observed for English.

**Conclusion:**

These findings provide a novel indication that the perceptual span is modified by the language being read to produce an asymmetry in the direction of reading and show for the first time that such an asymmetry occurs for reading Urdu.

## Introduction

Normal reading relies on the reader making a series of saccadic eye movements along each line of text, separated by periods of brief fixational pauses during which the eyes are relatively stationary and visual information is acquired from the text (for reviews, see [1], [2]). However, the region of text from which this information primarily is acquired on each fixational pause (usually called the perceptual span, [2]) is limited. Estimates of the size of the perceptual span have been obtained using a gaze-contingent eye-tracking paradigm in which changes are made to text dependent on the location of the reader's fixational pauses [3], [4]. This typically involves displaying a region of text as normal at the reader's point of fixation and obscuring text outside of this region (e.g., by replacing the original letters with xs). This region (or window) of normal text is moved in synchrony with the reader's eye movements, so that when the reader moves their eyes to fixate a new location, only text at this new location is shown normally and all other text is obscured. These changes are made sufficiently rapidly that the reader has the phenomenological experience that the window moves in perfect synchrony with their eye movements. The amount that this window of normal text extends to the right and left of fixation is usually varied across trials in an experiment and is informative about the amount of text that must be shown normally at the reader's point of fixation for reading performance to be normal.

Studies using this paradigm have been highly informative about the perceptual span for English and other alphabetic systems that are read from left to right, and have shown that skilled readers of English obtain useful information from an asymmetric area that extends approximately 14–15 character spaces to the right of fixation but no more than about 3–4 character spaces to the left [3]–[7] and typically no further than the beginning of the fixated word. These characteristics of the perceptual span for English (and other languages that are read from left to right) seem to survive over various viewing conditions (e.g., viewing distance and size of print [8], [9]). Consequently, it is generally accepted that, when reading from left to right, the perceptual span extends much further to the right of fixation than to the left.

The nature of the perceptual span is nevertheless generally considered to reflect the written characteristics of the language that is being read [10], [11]. In particular, it is argued widely that asymmetry in the perceptual span for alphabetic languages is related to the overall direction of reading. Accordingly, the perceptual span is asymmetrical to the right when reading English so that readers not only acquire information about the presently fixated word but also valuable information from parafoveal vision that might benefit the programming of saccadic eye movements that move the reader's point of fixation forward in text and benefit the pre-processing of words to the right of the current point of fixation [12]–[17].

However, while the perceptual span in English and other languages that are read from left to right has been widely studied (for a recent review, see [2]), the nature of the perceptual span for languages that are read from right to left has received much less attention. Indeed, arguments for the role of reading direction in determining asymmetry in the perceptual span are usually based on findings from a single study by Pollatsek, Bolozky, Well, and Rayner [18],which examined the perceptual span in Hebrew. In this study, native Israeli readers who were bilingual in Hebrew and English read sentences in which a window of normal text extended either 14 characters to the left of fixation and 4 characters to the right, or 4 characters to the left of fixation and 14 characters to the right. The findings showed that reading performance for Hebrew was superior when windows were asymmetric to the left whereas performance for English was superior when windows were asymmetric to the right. Thus, as Pollatsek et al. concluded, the overall direction of reading appears to modify the asymmetry of the perceptual span. However, this seminal study provided the sole evidence for more than 30 years that the perceptual span varies as a function of the direction of reading in an alphabetic language. Consequently, given the importance of this topic for understanding the processes involved in reading, and for implementing these processes in computational models of eye movement control (e.g., [19], [20]), there is considerable value in revealing more fully the nature of the perceptual span in languages read from right to left.

Indeed, recent research from our own laboratory with bilingual readers of Arabic (which also is read from right to left) and English [21] provided fresh support for the view that asymmetry in the perceptual span reflects the direction of reading. This research used a moving window paradigm in which a region of text was displayed as normal at the reader's point of fixation and text outside this region was obscured. The findings showed that when reading English, performance was superior when the windows of normal text were extended to the right of fixation. However, when reading Arabic, performance was superior when the windows extended to the left of fixation, and was essentially the reverse of that observed for English. These findings show that the perceptual span extends further to the left than to the right for Arabic, as well as Hebrew, and so provide a further demonstration that asymmetry in the perceptual span is determined by the direction of reading. The aim of the present research was to explore this influence of the direction of reading on the perceptual span of bilingual readers even more fully, by using a language other than Arabic or Hebrew that is read from right to left and that has characteristics that differentiate it from these other languages.

Accordingly, the present research examined the perceptual span in Urdu, which is also read from right to left. The writing system used for Urdu is derived from the Persian alphabet, which is similar to the Arabic alphabet, but is distinctive from the writing system used for both of these languages [22]. In particular, Urdu is traditionally written in an especially fluid calligraphic semi-cursive script (Nasta‘līq) in which the letters in words are often inter-linked and have variable widths and shapes depending on their location, and so may pose particular challenges to readers. Urdu is one of the two national languages of Pakistan, alongside English, and is widely used throughout Pakistan, Bangladesh, several regions of India, and amongst populations in Saudi Arabia, the UK, and USA. It is not uncommon for well-educated individuals, particularly those from Pakistan, to be bilingual in English and Urdu and to be able to read proficiently in both languages. An assessment of asymmetry in the region of text required by these bilinguals when reading in Urdu and English is therefore well-suited to revealing more fully the influence of reading direction on the perceptual span. Accordingly, and following the approach of Pollatsek et al. [18], we investigated the perceptual span of bilingual readers of Urdu and English using a version of the gaze-contingent moving-window technique used in previous research to control the amount of information available to the left and right of fixation during each fixational pause when English and Urdu is read.

However, rather than investigating the overall extent of the perceptual span, the focus of the present experiment was the influence of information within an area extending 2.5° either side of fixation. The reason for this approach is that the perceptual span encompasses a range of different types of information (e.g., inter-word spaces, word shape, letter identities; for a review, see [2]), broadly reflecting retinal eccentricity. Indeed, previous research using English suggests that letter identification during reading extends to only 8 or 9 characters to the right of fixation (equal to approximately 2.5° of visual angle under normal reading conditions) and just 4 characters (equal to approximately 1°) to the left (e.g., [7], [23]). But asymmetry in this area (which we call the central perceptual span) appears to be particularly influential for reading because it provides information which is required to identify the fixated word and, crucially, important information about the following word which aids the programming of saccadic eye movements and parafoveal pre-processing. Consequently, given its importance, the central perceptual span is well-suited to reveal contrasting directional asymmetries when reading Urdu and English and so the present study investigated the influence of this area, extending 2.5° to either side of fixation.

If asymmetry in the central perceptual span is determined primarily by the direction of reading in a language, Urdu should show a directional asymmetry that is essentially opposite to that for English. However, the situation for languages that are read from right to left may be even more complex and, given the particular characteristics of its writing system, this may be especially the case for Urdu. It is well-established that information presented to the left and right sides of each retina outside of foveal vision projects to each contralateral hemisphere (for reviews, see [24]–[27]). Thus, Urdu words presented to the left of central vision will project to the right hemisphere, and words to the right will project to the left hemisphere. Previous research has shown that word presentations to the right of central vision produce perceptual superiority due to left hemisphere dominance for language (for reviews, see [25], [26]). This hemispheric asymmetry in word processing is well-established for alphabetic languages such as English that are read from left to right, and studies have shown a similar asymmetry in alphabetic languages read from right to left, including Urdu [28]–[35], [37], [38]. Consequently, while rightward asymmetry in the perceptual span for English is consistent with greater use of information that projects initially to the dominant left hemisphere, a reversal of this asymmetry for Urdu would indicate greater use of information projected to the right (non-dominant) hemisphere, and this may be highly disadvantageous for reading (for further discussion, see [31], [32], [34]).

As a result, the influence of hemispheric dominance and reading environment provide different predictions for the asymmetry of the central perceptual span in languages such as Urdu that are read from right to left. If asymmetry in the central perceptual span is determined primarily by hemispheric projections, both Urdu and English should produce a rightward asymmetry. By comparison, if the central perceptual span is determined primarily by the direction of reading, there should be a rightward asymmetry in the central perceptual span when reading English but a leftward asymmetry when reading Urdu. If evidence of this shift in asymmetry were obtained it would provide a further indication, in line with findings from Arabic and Hebrew, that the asymmetry of the central perceptual span is modified by the overall direction of reading, and show for the first time that a leftward asymmetry occurs for Urdu.

**Figure 1:**
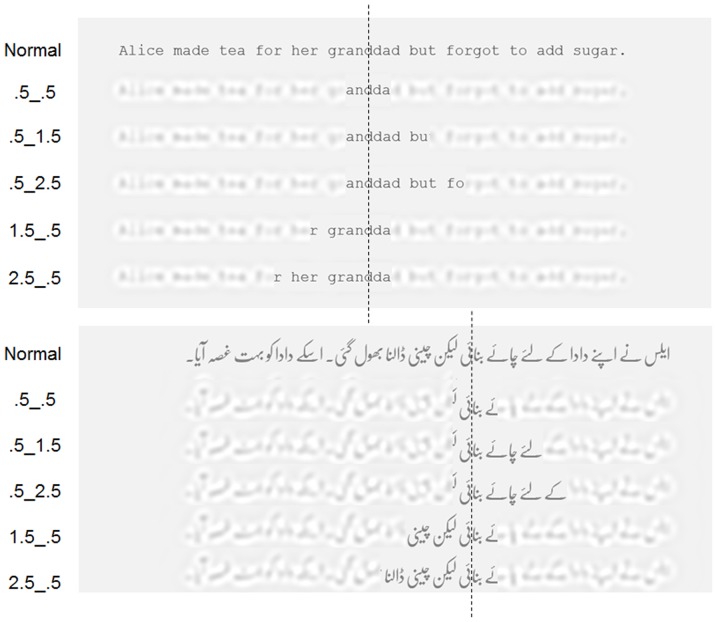
Examples of Urdu and English sentences displayed entirely as normal and with text falling within central vision shown as normal while text outside is filtered (see Method section). In these examples, the location of the fixation is denoted by the vertical dashed line. Note that the visual appearance of the filtered text in the figure is approximate due to restrictions in resolution and print medium.

To investigate this issue, we used a version of the gaze-contingent moving window technique that differed from that used in previous research [3], [4], [18]. Since its inception, the general principle of this paradigm has been that text within a specified window around the point of fixation is shown normally and text outside this window is obscured. In previous research, obscuring text outside the window has typically been achieved by replacing each letter with an x or another letter in order to obscure letter identities while preserving other characteristics of the text. However, while this approach is suitable for manipulating the perceptual span in printed English, where each individual character is spatially separate and distinct from its neighbors and has a constant width within a typeface, the same approach is not suitable for printed Urdu, because of the highly fluid, semi-cursive calligraphic typeface (Nasta ‘līq) in which the language is printed, and the extent to which letters in words are often inter-linked and have variable widths and shapes depending on their location. Because of these characteristics, letter replacements would grossly perturb the normal format of printed Urdu text and produce changes that were artefactually different from those produced for English. Accordingly, the present study avoided these problems by using visual filters to impair the visibility of letters outside each specified window whilst leaving the original letter content of both languages unchanged. The dimensions of these moving windows were systematically manipulated using five types of windows that varied how far the window of normal text (in degrees of visual angle) extended to the right and left of fixation. These windows extended either symmetrically 0.5° each side of fixation (.5_.5 windows), or asymmetrically 1.5° to the left and 0.5° to the right (1.5_.5 windows), 2.5° to the left and 0.5° to the right (2.5_.5 windows), 0.5° to the left and 1.5° to the right (.5_1.5 windows), or 0.5° to the left and 2.5° to the right (.5_2.5 windows). Within each language, 2.5° encompassed approximately 9 letters, and so the maximum extent of the asymmetric window to the left or right of fixation approximated the span over which letter identities have been shown previously to be established when reading English [7], [23]. Figure 1 provides an illustration of the displays created for English and Urdu using the visual filters and the various sizes of window used in the experiment.

## Results

**Figure 2:**
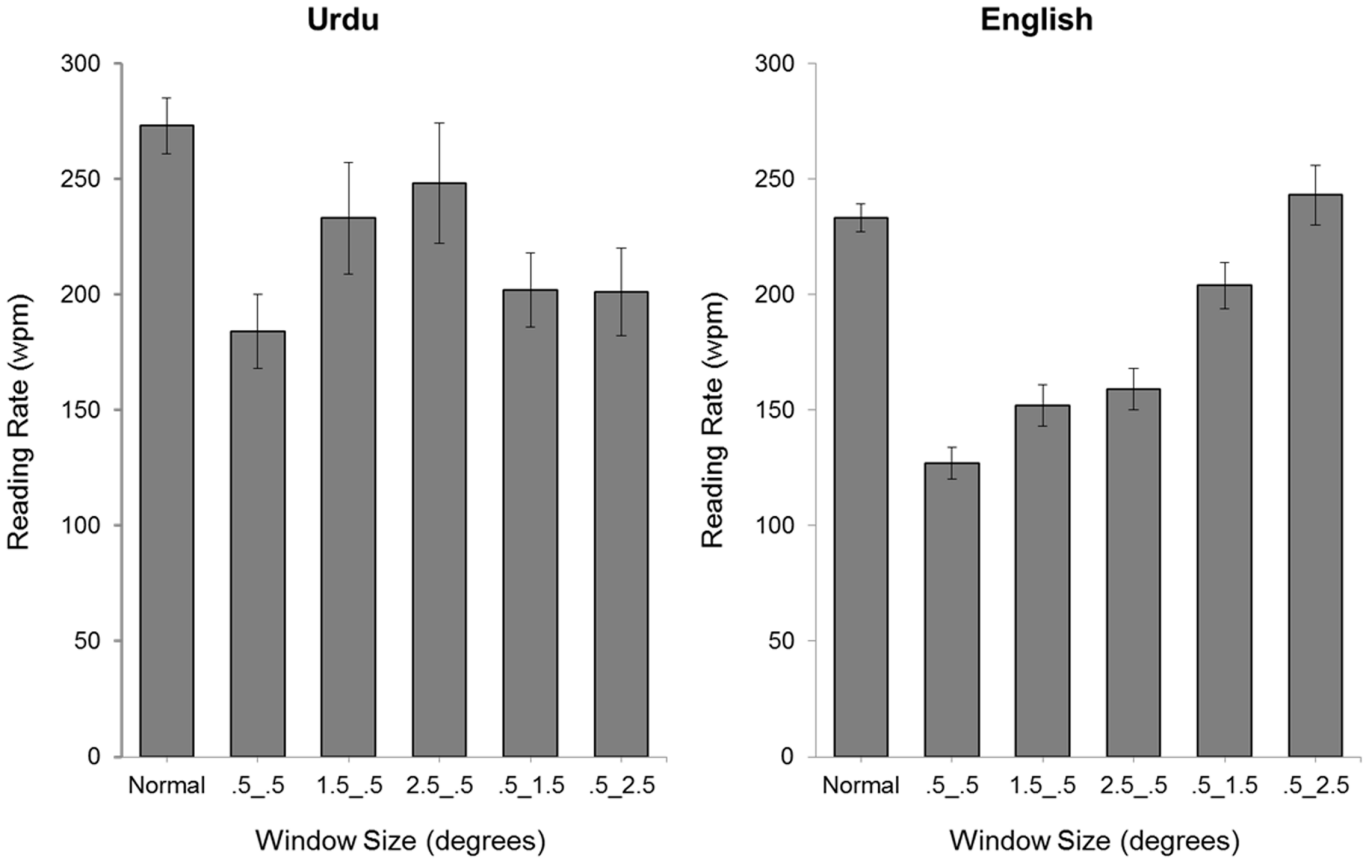
Mean reading rates (words per minute) for each display condition.

A range of eye movement measures was computed to assess the general characteristics of readers' eye movement performance [2]. Reading rates (calculated as words per minute) are shown in Figure 2, and fixation duration (the average length of fixational pauses during reading), number of fixations (the number of these fixational pauses), number of regressions (backwards movements in the text, i.e., to the right in Urdu and the left in English; including both inter- and intra-word regressions), and progressive saccade length (the amplitude, in degrees of visual angle, of forward movements in text, i.e., saccades to the left in Urdu and the right in English) are reported in Table 1. For each measure, effects of language (English, Urdu) and display condition (normal plus 5 window types) were analyzed using a repeated measures Analysis of Variance, computing error variance over participants (*F*_1_) and stimuli (*F*_2_). We also report partial eta-squared (η_p_^2^) as a measure of effect size based on the proportion of variance in the dependent variable that is attributable to our experimental manipulation. Pairwise comparisons were performed using a Bonferroni-corrected *t*-test (adjusted *p*<.05 for all significant effects). All pair-wise differences reported below were significant at this level.

**Table 1:** Eye Movement Measures for Each Display Condition in Urdu and English.

	Display Condition					
	Normal	.5–.5	1.5–.5	2.5–.5	.5–1.5	.5–2.5
Urdu						
Average Fixation Duration	272 (12)	303 (11)	276 (11)	274 (13)	294 (12)	290 (10)
Number of Fixations	20.2 (1.3)	23.9 1.4)	21.1 (1.2)	20.1 (1.2)	23.2 (1.7)	23.7 (1.9)
Progressive Saccade Length	2.2 (.3)	2.2 (.2)	3.0 (.3)	2.7 (.3)	2.3 (.3)	2.0 (.1)
Number of Regressions	2.5 (.5)	2.7 (.5)	1.7 (.2)	2.0 (.3)	2.3 (.4)	2.7 (.5)
English						
Average Fixation Duration	233 (6)	297 (9)	286 (8)	276 (7)	251 (8)	245 (8)
Number of Fixations	10.9 (.6)	19.1 (.8)	16.7 (1.1)	16.3 (.9)	13.8 (.6)	12.0 (.6)
Progressive Saccade Length	2.0 (.1)	1.6 (.1)	1.6 (.1)	1.6 (.1)	1.7 (.1)	1.8 (.1)
Number of Regressions	1.6 (.6)	3.2 (.8)	2.4 (.4)	2.3 (.3)	1.7 (.3)	1.6 (.2)

Durations are reported in milliseconds, saccade lengths are reported in degrees of visual angle, and Standard Errors are provided in parentheses.

### Reading Rates

Main effects were found for language, *F*_1_(1,11)  = 5.02, *p*<.05, η_p_^2^ = .31, *F*_2_(1,119)  = 44,36, *p*<.001, η_p_^2^ = .27, and display condition, *F*_1_(5,55)  = 16.71, *p*<.001, η_p_^2^ = .60, *F*_2_(5,595)  = 167.96, *p*<.001, η_p_^2^ = .59, together with an interaction of these factors, *F*_1_(5,55)  = 9.68, *p*<.001, η_p_^2^ = .47, *F*_2_(5,595)  = 52.17, *p*<.001 η_p_^2^ = .31. For Urdu, reading rates were lower than normal for all window conditions except for the 1.5_.5 and 2.5_.5 windows. By comparison, for English, reading rates were lower than normal for all window conditions except for .5_1.5, and .5_2.5 windows. Thus, the indication is that reading rates were normal for Urdu when the moving window extended asymmetrically to the left and normal for English when it extended asymmetrically to the right.

For Urdu, reading rates for the window conditions were lowest for .5_.5 windows, progressively higher for .5_1.5, and .5_2.5 windows, and highest of all for 1.5_.5 and 2.5_.5 windows. For English, reading rates for the window conditions were also lowest for .5_.5 windows, but equally higher for 1.5_.5 and 2.5_.5 windows, higher still for .5_1.5 windows, and highest for .5_2.5 windows. These findings therefore confirm that, in the moving window conditions, reading rates were fastest in Urdu when the windows were asymmetric to the left of fixation, and fastest in English when the windows were asymmetric to the right of fixation.

### Fixation Durations

The main effect of language was marginal for participants, *F*_1_(1,11)  = 3.67, *p*<.09, η_p_^2^ = .25, *F*_2_(1,119)  = 226.60, *p*<.001, η_p_^2^ = .66, but there was a main effect of display condition, *F*_1_(5,55)  = 45.99, p<.001, η_p_^2^ = .81, *F*_2_(5,595)  = 95.82, *p*<.001, η_p_^2^ = .45, together with an interaction of these factors, *F*_1_(5,55)  = 27.05, p<.001, η_p_^2^ = .71, *F*_2_(5,595)  = 35.58, *p*<.001, η_p_^2^ = .23. For Urdu, fixation durations were longer than normal for all window conditions except for those offset to the left of fixation (1.5_.5 and 2.5_.5 windows). Across windows, fixations for Urdu were longest for .5_.5 windows, shorter for .5_1.5 and .5_2.5 windows, and shortest for 1.5_.5 and 2.5_.5 windows. For English, fixation durations were longer than normal for all window conditions except for those offset to the right of fixation (.5_1.5 and .5_2.5 windows). Across windows, fixations for English were longest for .5_.5 windows, shorter for 1.5_.5 and 2.5_.5 windows, and shortest for.5_1.5 and .5_2.5 windows. Thus, the pattern of fixation durations resembled the pattern of reading rates and showed that fixation durations were shortest in the moving window conditions when windows were asymmetric to the left for Urdu and to the right for English.

### Number of Fixations

Main effects were found for language, *F*_1_(1,11)  = 24.98, *p*<.001, η_p_^2^ = .69, *F*_2_(1,119)  = 877.80, *p*<.001, η_p_^2^ = .88, and display condition, *F*_1_(5,55)  = 39.31, *p*<.001, η_p_^2^ = .78, *F*_2_(5,595)  = 62.32, *p*<.001, η_p_^2^ = .34, and there was an interaction of these factors, *F*_1_(5,55)  = 17.12, *p*<.001, η_p_^2^ = .61, *F*_2_(5,595)  = 22.73, *p*<.001, η_p_^2^ = .16. For Urdu, more fixations than normal were made for all window conditions and, across windows, most fixations were made for .5_.5, .5_1.5, and .5_2.5 windows, fewer fixations were made for 1.5_.5 windows, and fewest fixations were made for 2.5_.5 windows. For English, more fixations than normal were made for all window conditions and, across windows, most fixations were made for .5_.5 windows, fewer for 1.5_.5 and 2.5_.5 windows, fewer still for .5_1.5 windows, and fewest for .5_2.5 windows. The number of fixations therefore showed that fewer fixations were made in the moving window conditions in Urdu when windows were asymmetric to the left and in English when windows were asymmetric to the right.

### Progressive Saccade Length

There was a main effect of language, *F*_1_(1,11)  = 9.02, *p*<.05, η_p_^2^ = .45, *F*_2_(1,119)  = 3143.93, *p*<.001, η_p_^2^ = .96, but the main effect of display condition was not reliable by participants, *F*_1_(5,55) = 1.97, *p*<.10, η_p_^2^ = .15, *F*_2_(5,595)  = 76.35, *p*<.001, η_p_^2^ = .39. However, language and display condition interacted significantly, *F*_1_(5,55) = 6.03, *p*<.05, η_p_^2^ = .35, *F*_2_(5,595)  = 37.78, *p*<.001, η_p_^2^ = .39. For Urdu, saccades did not differ from normal for all windows except 1.5_.5, which produced saccades that were longer than normal. Across windows, saccades were equally longest for 2.5_.5 and 1.5_.5 and equally shorter for all other windows (i.e., .5_.5, .5_1.5, and .5_2.5 windows). For English, saccades were shorter than normal for all windows except .5_2.5. Across windows, saccades were longest for .5_2.5, shorter for .5_1.5, and equally shortest for all other windows. Progressive saccade length therefore provided an indication of a processing advantage for Urdu when windows were offset to the left, and for English when offset to the right.

### Number of Regressions

There was no main effect of language, *F*s<1.9, but a main effect of display type, *F*_1_(5,55)  = 3.10, *p*<.05, η_p_^2^ = .22, *F*_2_(5,595)  = 9.16, *p*<.001, η_p_^2^ = .07, and an interaction of these factors, *F*_1_(5,55)  = 4.28, *p*<.01, η_p_^2^ = .28, *F*_2_(5,595)  = 6.65, *p*<.001, η_p_^2^ = .05. For Urdu, the number of regressions was not different from normal for any windows and, across windows, 1.5_.5 produced fewer regressions than .5_.5, but no other effects were significant. For English, there were more regressions than normal for .5_.5, but regressions did not differ significantly from normal for any other windows. Across windows, most regressions were made for English for .5.5, fewer for 1.5_5 and 2.5_5, and fewest for .5_1.5 and .5_2.5. Number of regressions therefore revealed less clear influences of the moving windows on reading performance than other eye movement measures, but nevertheless showed evidence of a processing advantage for Urdu for windows offset to the left and for English for windows offset to the right.

## Discussion

The findings of the present study provide considerable evidence that the central perceptual span extends further to the left when reading Urdu and further to the right when reading English. It therefore seems clear that the asymmetry in the perceptual span for these languages is determined primarily by the direction of reading in the language. The evidence for this directional asymmetry was particularly evident in the reading rates for both languages, which did not differ compared to normal text when the moving window was extended in the direction of reading in either language, but were substantially longer for other windows. These results show that only a narrow region of normal text, encompassing no more than two or three degrees and offset in the direction of reading, is necessary for readers of Urdu and English to achieve normal reading rates.

Evidence of this directional asymmetry in both languages was apparent not only in reading rates but also in the duration and number of fixations made while reading, indicating that the asymmetry of the perceptual span in the direction of reading in the two languages influences both when and where the eyes move during reading. Although less informative than other measures, the frequency of regressive eye movements and the amplitude of progressive saccades also provide support for the existence of directional asymmetries in reading. In particular, readers made fewer regressions in the moving window conditions for Urdu when windows were offset to the left (significant only for 1.5_.5 windows) and for English when windows were offset to the right. Progressive saccades were also longer in Urdu when windows were asymmetric in the direction of reading, and a similar, albeit smaller, asymmetry in progressive saccade length was observed in English. These findings therefore indicate that the forward progression of the eyes through text was most efficient when moving windows extended asymmetrically in the direction of reading. In Urdu, progressive saccades were longer compared even to normal text when windows were asymmetric to the left of fixation. Consequently, it appears that extending the window to the left, while limiting the availability of text to the right, was particularly advantageous for saccade programming in Urdu, although further research is required to understand more fully how text to the left and right of fixation influences saccade programming in Urdu.

On the whole, the present findings resemble previous findings that for Hebrew showed a reversal in the asymmetry in the perceptual span for English [18], and more recent research showing a reversal in the perceptual span for Arabic [21]. The present findings therefore demonstrate that, for both Urdu and English, reading benefits from forward directed asymmetries that allow text to be previewed and provide valuable information about the location and identity of upcoming words. In English, his asymmetry has been shown to benefit the programming of saccadic eye movements and pre-processing of words in parafoveal vision to the right of fixation [12]–[17], and it now seems likely that similar parafoveal preview benefits are produced by text to the left of fixation in languages such as Urdu and Arabic that are read from right to left. Indeed, the indication from the findings for Urdu is that when these previews are not available (as in the window conditions of the present study), text during each fixation may be difficult to process and so reading rates become slower and fixations longer and more frequent.

However, while the directional asymmetry observed for English is consistent with previous indications concerning the perceptual span, the leftward asymmetry now observed for both Urdu and Arabic is of particular interest because of the processing disadvantages that unilateral right hemisphere projections are known to provide. In particular, not only is the right hemisphere generally non-dominant for language but evidence suggests that this hemisphere is particularly unsuitable for processing words written in Arabic [31], [32], [34]. Like Arabic, although differences exist, the writing system for Urdu uses a semi-cursive typeface and context-dependent letter shapes and so the right hemisphere may be similarly unsuited to processing words in this language. Nevertheless, as the directional asymmetry observed for Urdu in the present experiment, and for Arabic in our previous research [21], shows clear benefits for reading, as it produced shorter reading rates, and fewer and shorter fixations, it appears that this leftward asymmetry provides advantages for reading text in both languages that are not outweighed by disadvantages of right hemispheric projections.

Indeed, the existence of a leftward perceptual span for both Urdu and Arabic suggests that disruption produced by initial right hemisphere projections may be offset by other factors. Of particular importance is the indication that a region of central vision around fixation is likely to contain an overlap in hemispheric projections, so that information in this area projects to both cerebral hemispheres simultaneously (see [36]). The size of this region is unlikely to extend much more than about 1° either side of fixation but this may nevertheless help ameliorate disadvantageous effects of unilateral right hemisphere projections. Indeed, even areas away from fixation where information projects unilaterally to just one hemisphere may produce little or no functional division in processing between the two hemispheres because of rapid inter-hemispheric communication via the corpus callosum and sub-cortical connections [29], [36], [37].

A further source of influence may come from the nature of textual reading. In particular, although individual lateralized presentations of stimuli indicate that the right hemisphere is remarkably poor at processing words generally, textual reading provides a rather different environment in which forward-directed attentional processes and contextual and semantic cues can help enhance the processing of upcoming text (see [2]). In particular, considerable evidence from English, and other languages read from left to right, indicates that readers acquire low-level visual information about word boundaries and the physical extent of words in parafoveal vision during reading that may help pre-process word identities [12]–[17]. Consequently, although most of the visual field projects unilaterally to each contralateral hemisphere, the evidence from the present research of an influential leftward asymmetry when reading Urdu, and similar evidence from recent research with Arabic [21], indicates that the beneficial effects of a leftward perceptual span are not disadvantaged by right hemisphere projections.

It also was notable that, for both Urdu and English, performance advantages were sometimes observed when windows of normal text extended up to 2.5° in the opposite direction of reading (i.e., to the right for Urdu and to the left for English) in comparison with smaller symmetrical windows. The indication from this influence is that, for both languages, information from these areas benefitted reading and so may form part of the perceptual span during reading. One possibility is that this benefit for windows extending in the opposite direction of reading arises from the bilingual abilities of the participants taking part in our study. However, this finding also concurs with more recent evidence which suggests that the leftward component of the perceptual span for English is substantially larger than is widely assumed [38], [39]. Consequently, and in a similar vein, the present findings suggests that text to the right of the fixated word in Urdu contributes to reading performance, and therefore forms part of the perceptual span in this language.

The findings of the present study, therefore, provide a novel indication that the direction of asymmetry in the central perceptual span is modified by the overall direction of reading in a language and, for the first time, shows that a leftward asymmetry in the perceptual span occurs for Urdu. Thus, even though the writing systems of Urdu and English differ fundamentally in their visual and linguistic complexity, a common component of reading in each is the acquisition of information during each fixational pause from an area that extends asymmetrically in the direction of reading.

## Method

### Ethics Statement

This research was conducted with the ethical approval of the School of Psychology Ethics Committee at the University of Leicester, and in accordance with the ethical guidelines of the British Psychological Society. All participants gave informed consent in writing.

### Participants

Participants were 12 Urdu speakers from the University of Leicester. The majority of these participants were postgraduate students from Pakistan who had English as a second language at the level of proficiency required for postgraduate study. All participants were right handed, determined by a revised Annett Handedness Questionnaire [40], and had normal or corrected-to-normal visual acuity, determined by a Bailey-Lovie Eye Chart [41].

### Design and Materials

Stimuli were 120 Urdu sentences and 120 matched English sentences. Across languages, sentences were matched for numbers of words (Urdu, mean  = 11; English, mean  = 11) and characters (Urdu, mean  = 57, English, mean  = 59). Each sentence was shown entirely as normal or with normal text only within a window around the point of fixation. Five types of windows were used: 0.5° each side of fixation (.5_.5 windows), 1.5° to the left and 0.5° to the right (1.5_.5 windows), 2.5° to the left and 0.5° to the right (2.5_.5 windows), 0.5° to the left and 1.5° to the right (.5_1.5 windows), and 0.5° to the left and 2.5° to the right (.5_2.5 windows). Within each language, 2.5° encompassed approximately 9 letters. Text outside each window was obscured by using MATLAB to leave only spatial frequency content with a peak frequency of 2.2 cycles per degree (cpd) and low-pass and high-pass cut-off frequencies of 1.65–3.3 cpd [42], [43]. Custom software ensured that each window moved in close synchrony with eye movements. The phenomenological experience of participants was that each window moved in perfect synchrony with the eyes during reading.

All 240 sentences were randomized and sampled using a Latin square design so that each participant saw 20 different sentences in each of the 6 display conditions in each language. This ensured that all sentences were shown equally often in each condition in the experiment and that sentences in each language were shown only once to each participant. Urdu and English sentences were presented in separate sessions in a randomized order, and order of sessions was counterbalanced across participants. An additional 12 sentences were used as practice items at the beginning of each session.

### Apparatus and Procedure

Eye movements were recorded each millisecond using an Eyelink 1000 eye-tracker. Viewing was binocular, but only right eye movements were recorded. Sentences were displayed on a 19 inch monitor and eye position was sampled at 1000 Hz. Participants were instructed to read normally and for comprehension. At the start of the experiment, a 3-point horizontal calibration procedure was conducted, and calibration accuracy was checked before the presentation of each trial. At the start of each trial, a fixation square equal in size to a character space was presented to the right (for Urdu displays) or left (for English displays) of the screen. Once this was fixated, a sentence was then presented with its first letter at this fixation location. Participants pressed a response key once they finished reading each sentence. The sentence was replaced by a comprehension question that required an affirmative or negative response, which participants provided by pressing one of two response keys. Accuracy in answering these questions was greater than 90% for all participants for both English and Urdu, indicating that every participant showed a high level of comprehension in both languages.
